# Correlation Between *MYCN* Gene Status and MYCN Protein Expression in Neuroblastoma: A Pilot Study To Propose the Use of MYCN Immunohistochemistry in Limited-Resource Areas

**DOI:** 10.1200/JGO.19.00135

**Published:** 2019-07-31

**Authors:** Teresa Santiago, Nidale Tarek, Fouad Boulos, Caleb Hayes, Sima Jeha, Susana Raimondi, Carlos Rodriguez-Galindo

**Affiliations:** ^1^ St Jude Children's Research Hospital, Memphis, TN; ^2^ American University of Beirut Medical Center, Beirut, Lebanon

## Abstract

**PURPOSE:**

The most significant adverse risk factor for neuroblastoma (NB) is *MYCN* gene amplification, which strongly associates with high-risk disease. Fluorescent in situ hybridization (FISH) is considered the best method to evaluate *MYCN* gene status. However, it requires a laboratory that can perform highly complex testing, specialized personnel, and costly reagents. Herein, we aimed to investigate the feasibility of using immunohistochemistry (IHC) to detect MYCN protein expression in lieu of FISH, a strategy potentially useful in areas with limited resources.

**METHODS:**

A pilot cohort of 78 patients with NB, including 34 of Middle Eastern descent (MED) who had a higher prevalence of *MYCN* gene amplification (44.11%) and 44 of North American descent (NAD), nine (20.45%) of whom had *MYCN* amplification, was evaluated with IHC for MYCN protein. Correlations of FISH results and protein expression are presented.

**RESULTS:**

A positive correlation between *MYCN* gene amplification and protein expression by IHC was seen in 22 (91.66%) of the 24 *MYCN*-amplified NB cases—14 (93.33%) of 15 patients of MED and eight (88.88%) of nine patients of NAD. Agreement between negative FISH and negative IHC results was noted in 18 (94.73%) patients of MED and 34 (97.14%) patients of NAD. Two cases had weak protein expression but no gene amplification (MED: n = 1; 5.0%; NAD: n = 1; 2.9%).

**CONCLUSION:**

An excellent overall correlation between *MYCN* gene status by FISH and MYCN protein expression by IHC was confirmed. MYCN IHC in NB with reflexing to FISH in equivocal cases is potentially useful in a limited-resource setting. Evaluation of effectiveness using a larger cohort and optimization to perform MYCN IHC manually is needed.

## INTRODUCTION

*MYCN* gene amplification is considered the single most relevant genetic alteration in patients with neuroblastoma (NB) and is associated with advanced-stage disease, high-risk category, and poor prognosis.^1^ The overall incidence of *MYCN* gene amplification in patients with NB is approximately 19%, with recognized variations among different ethnic groups, such as 29% in Native American patients^2^ and 44% in patients of Middle Eastern descent (MED) .^3^ The methodology of choice to identify *MYCN* gene amplification is fluorescent in situ hybridization (FISH), which can be performed within a rather short turnaround time. However, the availability of the FISH assay is widely restricted to high-income countries owing to the cost of necessary equipment and reagents, and specialized personnel.

On the basis of current estimates, it is expected that more than 400,000 children are diagnosed with cancer worldwide every year.^4^ NB is considered the second most common pediatric solid neoplasm after the brain tumors, representing approximately 6% to 7% of all the pediatric neoplasms.^5^ Even though the incidence of NB appears to be increased in areas that demonstrate a higher human development index,^6^ approximately 24,000 to 28,000 new NB cases are expected per year globally, and a great proportion of them will occur in areas with limited resources. In this pilot study, we investigated the feasibility of using immunohistochemistry (IHC) assay to determinate MYCN protein expression level in patients with NB. We propose the use of the IHC assay as an alternative, cost-effective solution to further risk classify patients with NB in areas with limited resources where the FISH assay to determinate *MYCN* gene status is not readily available.

**CONTEXT**

**Key Objective**
Is it feasible to use immunohistochemistry (IHC) to detect MYCN protein expression instead of performing fluorescent in situ hybridization (FISH) to identify *MYCN* gene amplification in neuroblastoma (NB) in areas with limited resources?
**Knowledge Generated**
In a pilot cohort of 78 cases, we showed an excellent correlation between MYCN protein expression by IHC and *MYCN* gene amplification status by FISH (91.66% sensitivity and 96.29% specificity).
**Relevance**
MYCN IHC appears to be potentially useful in a limited-resource setting to aid in the risk classification of patients with NB. Any equivocal MYCN IHC results should be reflexed to *MYCN* gene FISH assay.

## METHODS

### Case Selection

Study approval was obtained from all participating institutions in compliance with the international regulations for protection of human research subjects. From our pathology file, 78 cases diagnosed as NB between July 2014 and July 2017 were selected. They included 34 patients submitted as consecutive pathology consultations for central review from our Middle East partner institution in Lebanon and 44 consecutive patients who were diagnosed and treated in our hospital. The available clinical data, histopathological features, and patient’s outcome were reviewed.

### Fluorescence In Situ Hybridization

All 78 cases were evaluated with FISH assay as part of the pathology review. Laboratory-developed probes targeting the *MYCN* gene (2p24) and a control *PAX3* probe (2q35) were used. The *MYCN* gene was considered amplified when the signals were equal or exceeded more than 10 copies in a given tumor cell nucleus and surpassed at least three times the number of the control signals. The entire tissue section or cytologic smears from bone marrow (BM) aspirates were examined, and at least 200 tumor cells were evaluated. The percentage of neoplastic cells showing gene amplification was documented. Cases with an equal number of *MYCN* signals and control signals, or with a difference less than three times the number of the control signals were interpreted as negative for *MYCN* gene amplification.

### IHC Analysis

At least one representative formalin-fixed, paraffin-embedded tissue section from each of the 78 cases was selected for the MYCN IHC assay. In brief, heat-induced epitope retrieval was conducted with a ready-to-use EDTA-based solution with pH of 9.0 (BOND Epitope Retrieval Solution 2; Leica, Newcastle, United Kingdom) and incubated for 20 minutes. Mouse monoclonal antibody anti-MYCN (Clone NCM II; catalog no. 16898100; Abcam, Cambridge, MA) was diluted at a 1:150 ratio with a ready-to-use Tris-buffer–based solution (BOND primary antibody diluent, catalog no. AR9352; Leica). All the staining steps were performed using a fully automated IHC stainer (BOND-MAX; Leica). Before examination by light microscopy, all the slides were counter-stained with hematoxylin. Any degree of nuclear staining (strong or weak) in any quantity (diffuse or focal) was categorized as positive and considered evidence of MYCN protein expression. Conversely, cases with no nuclear positivity were interpreted as negative and therefore scored as no indication of MYCN protein expression. All the IHC staining patterns were correlated with the *MYCN* FISH results.

## RESULTS

### Clinical Features, Histologic Findings, and FISH results

The main clinical features of all 78 patients are listed in Table 1. Overall, there was no statistically significant difference regarding sex. The youngest patient was diagnosed at 1 week of age and the oldest patient at 19 years of age (mean age at diagnosis, 37.77 months). Most cases were primarily from the adrenal gland (41.03%), and metastatic disease was identified in 45 patients (stage IV, 57.69%). For some patients, the original diagnosis was based on BM involvement. Outcome information is available for 67 patients (85.89%). Seven patients of MED were not treated at our Middle East partner institution; therefore, no additional information was available (pathology consultation only). Four other patients of MED were lost to follow-up.

**TABLE 1:**
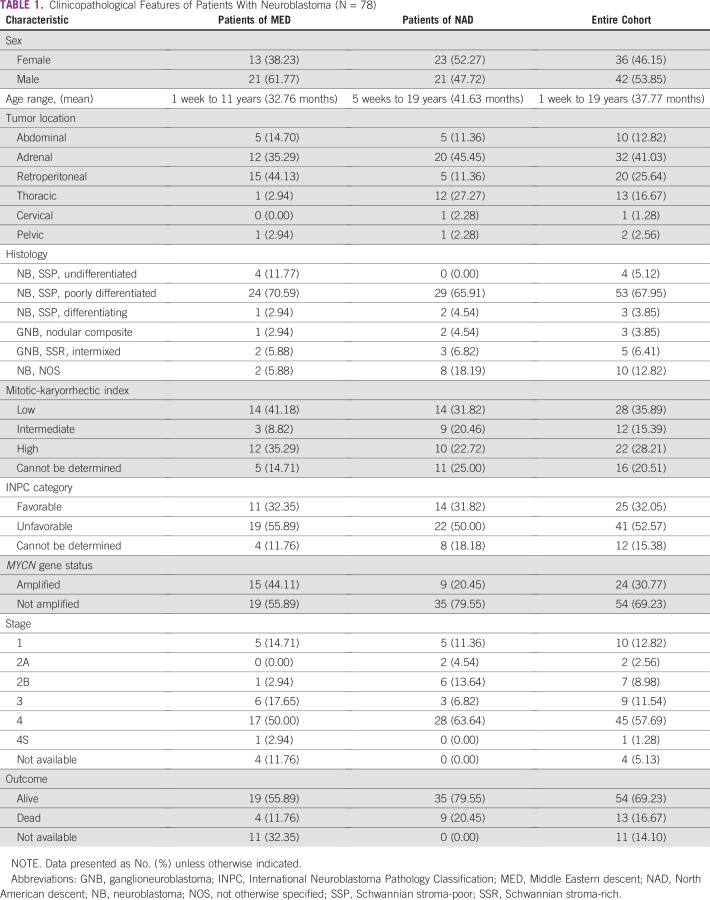
Clinicopathological Features of Patients With Neuroblastoma (N = 78)

Table 1 also lists relevant histopathological characteristics such as tumor histology, mitotic-karyorrhectic index (MKI), International Neuroblastoma Pathology Classification (INPC) category, and *MYCN* gene status evaluated by FISH. As previously reported by our group, a higher incidence of *MYCN* gene amplification was noted in the patients of MED when compared with the NAD group (44.11% *v* 20.45%, respectively).^3^ Among the 24 *MYCN*-amplified cases in our cohort (24 of 78), the percentage of neoplastic cells showing evidence of *MYCN* gene amplification in a given tumor ranged from 18.5% to 100%, with an average of 87.89%.

### IHC Findings of MYCN Protein

**FIG 1:**
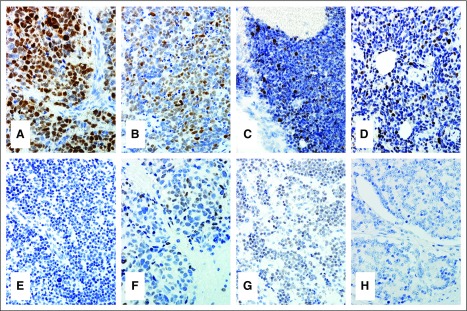
MYCN protein immunohistochemistry (IHC) in neuroblastoma (NB). (A-D) Four *MYCN*-amplified NB cases showing different degree and intensity of MYCN protein expression by IHC. (E) An NB case negative for both *MYCN* gene amplification and protein expression. (F, G) Two NB cases negative for *MYCN* gene amplification but displaying weak immunoreactivity. (H) *MYCN*-amplified NB but negative IHC staining.

The correlation between the IHC and FISH results are listed in Table 2. Overall, MYCN protein expression by IHC was seen in 22 (91.66%) of the 24 *MYCN*-amplified NB cases. The number of positive cells and the intensity of the nuclear staining were heterogeneous among the cases. Diffuse and strong nuclear staining were observed in some cases, whereas others had limited expression with only a few but unequivocally positive nuclei (Figs 1A-1D).

**TABLE 2:**
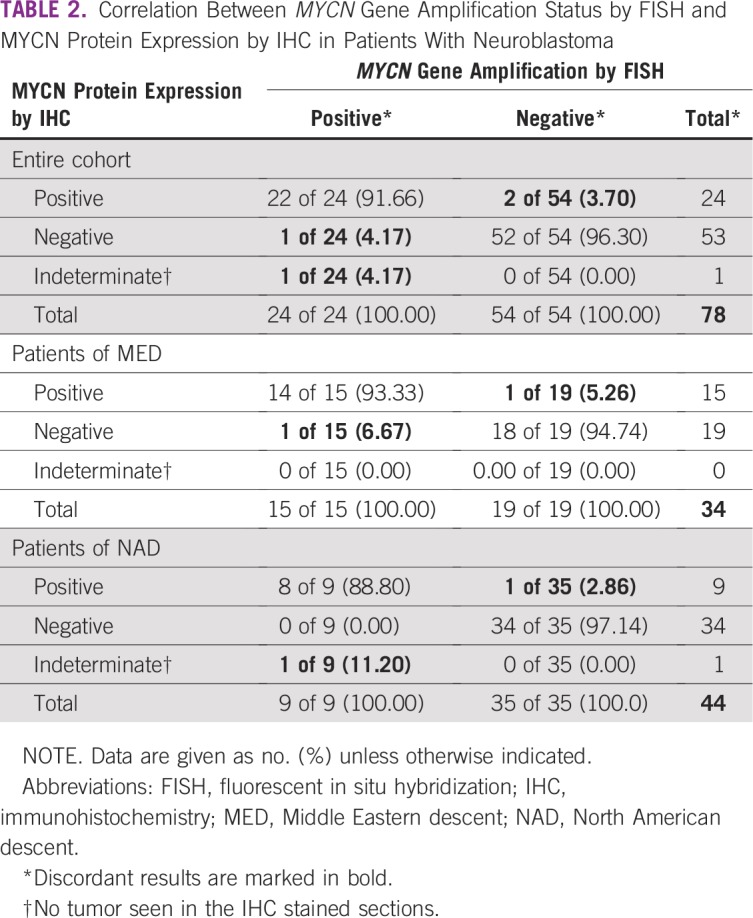
Correlation Between *MYCN* Gene Amplification Status by FISH and MYCN Protein Expression by IHC in Patients With Neuroblastoma

One patient of MED with *MYCN* amplification had a discordant result and lacked evidence of protein expression (Fig 1H). In one patient of NAD with stage IV disease and BM involvement by NB at diagnosis, the FISH assay was performed on the BM cytologic smears and interpreted as positive for amplification. IHC staining was attempted in the postchemotherapy resection specimen, but no residual tumor was identified. Therefore, the MYCN protein expression, in this case, was considered indeterminate.

Agreement between negative FISH and negative IHC was noted in 52 (96.30%) patients—19 (94.73%) patients of MED and 34 (97.14%) patients of NAD. One *MYCN*-nonamplified case from each group had weak and diffuse (1+) staining (Figs 1F and 1G). In all the three cases with discordant results, the staining for MYCN IHC was repeated at least two times. The sensitivity and specificity of the MYCN IHC assay was 91.66% (95% CI, 71.52 to 98.54) and 96.29% (95% CI, 86.16 to 99.35), respectively.

### Correlation Between MKI, *MYCN* Gene Status by FISH and MYCN Protein Expression by IHC

**FIG 2:**
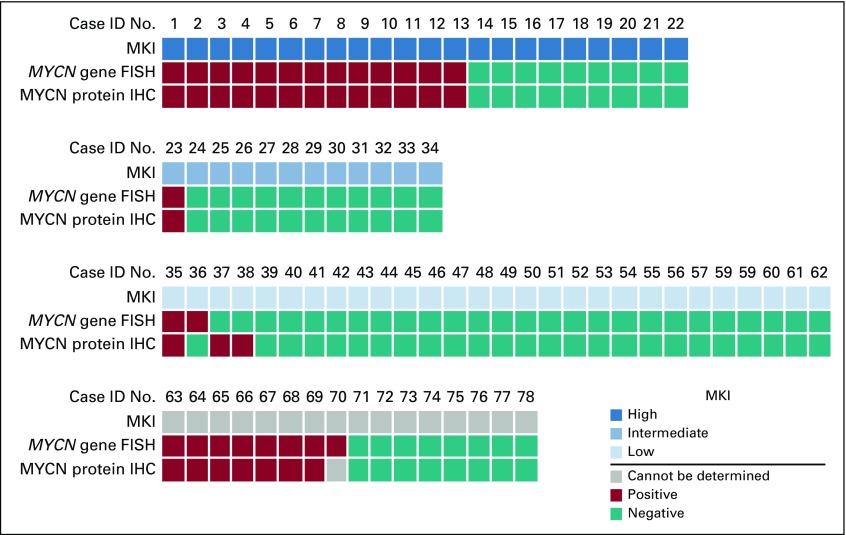
Correlation between *MYCN* gene amplification status by fluorescent in situ hybridization (FISH) and MYCN protein expression by immunohistochemistry (IHC) in patients with neuroblastoma according to the mitotic and karyorrhectic index (MKI). ID, identification.

On the basis of MKI, 22 cases (28.21%) were classified as high MKI, 12 (15.38%) were intermediate MKI, and 28 (35.90%) were low MKI. For 16 cases (20.51%), MKI was not assigned, due to the nature of the sample (BM biopsy or post-therapy specimen) or crushing artifact. Thirteen of 22 (59%) of high-MKI NB had *MYCN* gene amplification by FISH and concordant MYCN protein nuclear overexpression by IHC. In fact, all 22 high-MKI cases had a concordant result between FISH and IHC (Fig 2). Among the 12 cases graded with intermediate MKI, only one (8.33%) had both *MYCN* gene amplification and MYCN protein expression. The low-MKI group had the lowest incidence of *MYCN* gene amplification (two of 28; 7.14%) and included all three discordant cases. Fifteen of 16 cases (93.75%) in which MKI was not assigned had consistent results between FISH and IHC. As previously mentioned, one *MYCN*-amplified case in which the FISH assay was performed on the diagnostic BM cytologic smear (and MKI was not scored) did not show any viable residual tumor in the available post-therapy resection specimen. Therefore, the correlation between FISH and IHC could not be established in this case.

## DISCUSSION

More than 20 years ago, FISH was described as an alternative method to detect *MYCN* gene amplification in NB in place of Southern blot analysis.^7^ Subsequently, the presence of *MYCN* gene amplification was recognized as being associated with an aggressive clinical course in patients with NB^8^ and therefore required for the risk classification and treatment selection of these patients.^1^ Still, the availability of FISH assay remains generally restricted to developed countries. Many of the NB cases we receive for pathology review from international institutions, including the ones referred from our Middle East partner center in Lebanon, are submitted mostly because they are unable to determine the *MYCN* gene status locally. The high cost to implement and maintain a specialized FISH laboratory and the lack of trained personnel are usually the limiting factors. More recently, investigators from the Children’s Oncology Group had analyzed 241 NB cases with high MKI; among the 120 *MYCN*-amplified high-MKI cases, 101 (84.2%) showed evidence of positive MYCN staining and were associated with worse 4-year event-free survival.^9^ In fact, the majority of the MYCN-amplified NBs express MYCN protein.^10^ However, there is also evidence that overexpression of MYCN protein can happen in cases without gene amplification and similarly appears to act as an independent poor prognostic factor.^11^

In this pilot study, we investigated how well, in our hands, the expression of MYCN protein by IHC would correlate with our FISH results in samples collected and processed in our hospital, as well as in referred specimens from one of our international partner sites. The possibility of using IHC in lieu of the FISH assay in cases of NB seems to be a potentially useful strategy and cost-effective solution in a resource-limited setting, particularly in regions of the world where the incidence of *MYCN-*amplified NB is higher, such as in the Middle East.^3^ Furthermore, the potential influence of the patient’s genetic background on the phenotype of their tumors made us choose to investigate the correlation between the *MYCN* gene FISH results and IHC protein expression levels in two ethnically distinct groups of patients (MED and NAD). However, there was no statistically significant difference between them.

Although the overall correlation between a positive FISH assay and positive IHC testing was excellent (91.66%), the degree and level of intensity of the MYCN protein expression were diverse. Some cases with 100% of the analyzed cells by FISH with more than 10 copies of the gene displayed diffuse and strong immunoreactivity (Fig 1A), whereas others with a similar degree of gene amplification exhibited weaker or only focal nuclear positivity by IHC (Figs 1B-1D). This fact emphasizes the importance of IHC reaction optimization and interpretation. Careful analysis of the entire slide is critical. Nonetheless, previous studies have suggested that cases with *MYCN* gene amplification and diffuse and strong protein expression would correlate with a worse outcome when compared with cases with *MYCN* gene amplification but only focal positivity or negative MYCN IHC.^9^ Presently, only the *MYCN* gene status by FISH (not the protein expression level) is considered in the risk classification of patients with NB.

There was no statistical difference between patients of MED and NAD regarding the correlation between *MYCN* amplification status and MYCN protein expression level. Only three cases in our entire cohort of 78 (3.84%) demonstrated discordant results. One was an 11-year-old Lebanese boy who had a large retroperitoneal mass diagnosed as NB, Schwannian stroma-poor, poorly differentiated with low MKI but classified as unfavorable histopathology on the basis of INPC. FISH targeting the *MYCN* gene showed focal amplification in 18.5% of the tumor cells analyzed. This patient was treated per the high-risk category and was alive with no evidence of disease at his last follow-up visit. MYCN IHC was performed twice in this case, and no evidence of MYCN protein expression was noted. It is possible that the tissue sections used for the IHC (although from the same paraffin block used for FISH) did not include the tumor component that harbors the amplification, or, even though the *MYCN* gene was amplified, the tumor cells, indeed, did not express the protein.

The other additional discordant results were two *MYCN* gene nonamplified cases that showed weak diffuse nuclear staining (IHC staining was repeated twice). One was the case of a 5-year-old Lebanese girl diagnosed with a stage 3 (abdominal mass) Schwannian stroma-poor, poorly differentiated, low-MKI NB (unfavorable INPC, high-risk category) who was alive at the last follow-up. The second case was that of a NAD female patient diagnosed with localized, thoracic, Schwannian stroma-poor, poorly differentiated, low-MKI NB (favorable INPC) who was alive and well at her follow-up. The association of undifferentiation or poorly differentiated morphology and high MKI with *MYCN* gene amplification in NB is well known. Interestingly, both cases had a low proliferative index and low MKI, which make us speculate that the weak IHC positivity could be due to very low levels of protein expression secondary to a mechanism other than gene amplification, or to just background staining. Nevertheless, it is our recommendation to reflex to FISH assay any case that demonstrates questionable or weak MYCN IHC staining.

When we examined our cohort on the basis of MKI (Fig 2), as expected, the highest percentage of *MYCN*-amplified cases were included in the high-MKI category (13 of 22; 59%) and no discrepancy between FISH and IHC results was observed in this subgroup. Only one of 12 (8.33%) intermediate-MKI case and two of 28 (7.14%) low-MKI cases had both *MYCN* gene amplification and MYCN protein expression. The low-MKI group, indeed, had the lowest incidence of *MYCN* gene amplification (7.14% low MKI *v* 59% high MKI; *P* < .01) and included all three discordant cases—the two previously discussed cases that had weak IHC staining but no gene amplification, and the one *MYCN*-amplified NB that lacked protein expression. Except for one case in which no viable residual tumor was available in the post-therapy resection specimen to perform MYCN IHC, all the remaining 15 cases (93.75%) in which MKI could not be assigned had concordant results (FISH positive/IHC positive, n = 7; FISH negative/IHC negative, n = 8).

The FISH assay is considered the gold standard method of investigating *MYCN* gene status in patients with NB, but the upfront investment to implement a FISH laboratory versus introducing the IHC assay to an already-established anatomic pathology laboratory is considerably higher and, unfortunately, not feasible in many places. Whereas most laboratories now perform automated IHC, this assay, when well validated and applying stringent optimization, can be performed manually with good results. Considering the time, effort, complexity, and direct expenses, the final technical and interpretative cost of an individual FISH test or an IHC staining can vary from country to country, and even within the same country, affected by local or regional factors. A comparison using the potential basic reimbursement offered by Medicare (or other third-party payers in the United States), based on the American Medical Association Current Procedural Terminology (CPT) coding, foresees a higher estimated fee for a FISH test compared with IHC: The cost of FISH, calculated as CPT 88271 times two probes plus CPT 88275 for interpretation of 100 to 300 cells plus CPT 88291 for reporting, is approximately $132.00; the cost of IHC, calculated as CPT 88342 technical component plus professional component, is approximately $65.00. All values are rounded up and expressed in US dollars without applying local modifiers or conversion factors. Although these estimated fees may not represent the actual cost of these tests, this analogy emphasizes that using IHC instead of the FISH could be a cost-effective solution for low- and middle income countries. In Lebanon, for instance, the price difference between a FISH assay and one IHC stain is also substantial (one FISH test costs approximately $300.00 *v* one IHC, which costs approximately $100.00).

In summary, on the basis of the findings of this pilot analysis, we believe the use of MYCN IHC could be a good, cost-effective strategy to aid the diagnosis and classification of NB cases in areas where the implementation of FISH would be costly and technically challenging. Evaluation of a larger cohort of cases and, most importantly, optimization to perform MYCN IHC reaction manually, is still required.
